# Immunomodulatory and Regenerative Functions of MSC-Derived Exosomes in Bone Repair

**DOI:** 10.3390/bioengineering12080844

**Published:** 2025-08-05

**Authors:** Manorathna Arun, Sheeja Rajasingh, Parani Madasamy, Johnson Rajasingh

**Affiliations:** 1 Department of Genetic Engineering, School of Bio-Engineering, SRM Institute of Science and Technology, Kattankulathur, Chengalpattu 603203, Tamil Nadu, India; ma1987@srmist.edu.in (M.A.); paranim@srmist.edu.in (P.M.); 2 Department of Bioscience Research, University of Tennessee Health Science Center, Memphis, TN 38163, USA; srajasin@uthsc.edu; 3 Department of Microbiology, Immunology, and Biochemistry, University of Tennessee Health Science Center, Memphis, TN 38163, USA; 4 Department of Medicine—Cardiology, University of Tennessee Health Science Center, 847 Monroe Avenue, Memphis, TN 38163, USA

**Keywords:** mesenchymal stromal cells, exosomes, osteocytes, immunomodulation, bone regeneration

## Abstract

Bone integrity is maintained through continuous remodeling, orchestrated by the coordinated actions of osteocytes, osteoblasts, and osteoclasts. Once considered passive bystanders, osteocytes are now recognized as central regulators of this process, mediating biochemical signaling and mechanotransduction. Malfunctioning osteocytes contribute to serious skeletal disorders such as osteoporosis. Mesenchymal stromal cells (MSCs), multipotent stem cells capable of differentiating into osteoblasts, have emerged as promising agents for bone regeneration, primarily through the paracrine effects of their secreted exosomes. MSC-derived exosomes are nanoscale vesicles enriched with proteins, lipids, and nucleic acids that promote intercellular communication, osteoblast proliferation and differentiation, and angiogenesis. Notably, they deliver osteoinductive microRNAs (miRNAs) that influence osteogenic markers and support bone tissue repair. In vivo investigations validate their capacity to enhance bone regeneration, increase bone volume, and improve biomechanical strength. Additionally, MSC-derived exosomes regulate the immune response, creating pro-osteogenic and pro-angiogenic factors, boosting their therapeutic efficacy. Due to their cell-free characteristics, MSC-derived exosomes offer benefits such as diminished immunogenicity and minimal risk of off-target effects. These properties position them as promising and innovative approaches for bone regeneration, integrating immunomodulatory effects with tissue-specific regenerative capabilities.

## 1. Introduction

Maintaining skeletal integrity and mineral homeostasis is crucial for overall health, as disruptions in these processes underlie a range of debilitating bone disorders. Central to this dynamic balance is bone remodeling, a continuous and tightly regulated physiological process involving the coordinated activity of osteocytes, osteoblasts, and osteoclasts [1]. Among these, osteocytes, the most abundant cell types in bone tissue, play a pivotal role in regulating both bone formation and resorption. Once considered passive, osteocytes are now recognized as central orchestrators of bone remodeling, functioning through biochemical signaling and mechanotransduction [2]. In response to mechanical and biochemical cues, osteocytes secrete substances such as sclerostin, which inhibits bone growth, and prostaglandins and IGF-1, which promote bone growth [3,4]. Their extensive dendritic networks allow for intercellular communication across the bone matrix, ensuring synchronized remodeling activity [5]. Mechanistically, osteocytes regulate osteoblast and osteoclast function via key signaling pathways, including Wnt/β-catenin and the OPG/RANKL axis [6,7].

The proper function of osteocytes is essential for preserving bone microarchitecture and systemic mineral homeostasis, especially for calcium and phosphate regulation [7]. Dysregulation of osteocyte activity contributes to a spectrum of skeletal pathologies, such as osteoporosis and high bone mass syndrome. While significant advances have been made in understanding osteocyte biology, many regulatory mechanisms remain to be fully understood, highlighting the need for continued research and the integration of emerging technologies. To address these challenges, regenerative strategies have increasingly turned toward mesenchymal stromal cells (MSCs) and their secreted extracellular vesicles, particularly exosomes. MSCs are multipotent cells capable of osteogenic differentiation and which exert potent immunomodulatory effects [8]. Their therapeutic potential has been demonstrated across a range of inflammatory and degenerative conditions [9]. Notably, it is now widely recognized that MSCs exert many of their regenerative effects through exosomes—nanosized vesicles rich in proteins, lipids, and nucleic acids that facilitate paracrine signaling [10]. MSC-derived exosomes are key mediators at the intersection of bone regeneration and immune modulation. They promote osteoblast proliferation and differentiation, deliver osteoinductive microRNAs, and promote angiogenesis [11,12,13]. Additionally, they influence immune cell behavior by modulating cytokine release and promoting anti-inflammatory phenotypes [14,15,16]. These vesicles enhance regeneration, inhibit apoptosis, and stimulate angiogenesis in multiple disease models, making them an attractive cell-free therapeutic tool for cancer [17,18] and spinal cord injury [19].

Emerging evidence also emphasizes the critical role of immunomodulation in bone homeostasis, particularly in osteocyte differentiation and function. The bidirectional communication between immune cells and bone-resident cells, a concept central to osteoimmunology, shapes the bone remodeling landscape. Cytokines such as IL-6, TNF-α, and RANKL, produced by immune cells, directly influence osteoclast and osteoblast activity [20]. Both innate and adaptive immune cells, including macrophages, dendritic cells, T cells, and B cells, contribute to this tightly regulated interface [21]. MSC-derived exosomes further contribute to this immunomodulatory network. For instance, inflammatory preconditioning of MSCs can enhance the immunomodulatory content of exosomes, promote anti-inflammatory macrophage polarization, and favor osteogenesis while limiting bone resorption [22]. While balanced immune signaling supports osteocyte differentiation and bone regeneration, excessive or dysregulated immune activation can result in pathological bone loss, emphasizing the importance of immune homeostasis in skeletal health [23].

## 2. Mesenchymal Stromal Cells and Exosomes: An Overview

**Figure 1:**
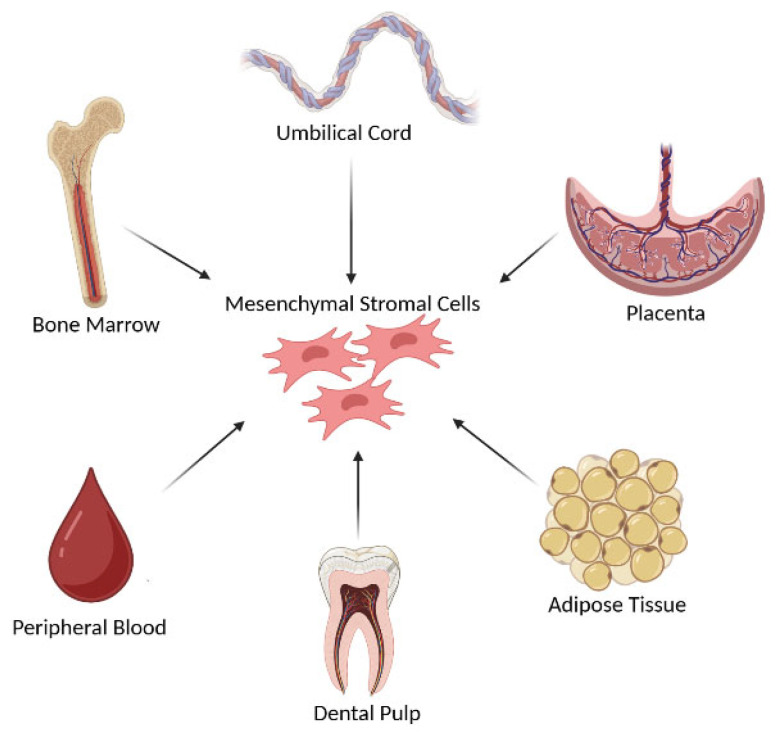
**Sources and differentiation potential of mesenchymal stem cells (MSCs).** Mesenchymal stem cells (MSCs) can be isolated from various tissue sources, including bone marrow, adipose tissue, the umbilical cord, placenta, dental pulp, and peripheral blood. These multipotent cells possess the ability to differentiate into a range of cell types such as osteocytes, chondrocytes, adipocytes, myocytes, neuronal cells, and tenocytes. Additionally, MSCs play roles in regenerating tissues of the lungs, kidneys, liver, and vasculature, highlighting their broad therapeutic potential in regenerative medicine.

MSCs are well recognized for their extensive proliferation capacity and trilineage differentiation ability into osteocytes, adipocytes, and chondrocytes [24,25]. Although historically referred to as “mesenchymal stem cells,” it is now understood that isolating and enriching a functionally validated stem cell subset from this heterogeneous population remains a significant challenge. In the absence of substantial in vivo evidence and appropriate markers to identify true stem cell traits, the term mesenchymal stromal cells (MSCs) more accurately reflects this cell population’s known biology and function. Notably, the primary confirmed in vivo role of MSCs is to establish a supportive microenvironment for other stem cells [26]. In vitro, MSCs are characterized by their adherence to plastic surfaces and the expression of specific cell-surface-positive markers such as CD73, CD90, and CD105, and negative markers CD45 and CD34 [27]. In addition to their multilineage differentiation potential, MSCs exhibit significant immunomodulatory properties and robust self-renewal capabilities, making them highly suitable for a wide range of therapeutic applications due to their accessibility and expandability [24,25]. MSCs can be isolated from various sources, broadly categorized into adult, perinatal, and dental tissues (Figure 1). Common adult sources include bone marrow MSCs (BM-MSCs) and adipose tissue MSCs (AD-MSCs). Perinatal sources, such as umbilical cord blood and Wharton’s, offer MSCs with superior proliferative potential and lower immunogenicity [28]. Dental-derived MSCs, such as those from the dental pulp, have recently gained interest for applications in regenerative dentistry [29,30]. These cells play an important role in the regeneration of craniofacial structures and are being investigated for the treatment of congenital malformations, trauma-related defects, and oral diseases [31,32]. Furthermore, dental MSCs are essential for the regeneration of periodontal supporting tissues such as alveolar bone, periodontal ligament, and cementum—particularly in the treatment of periodontitis [33].

Beyond direct MSC transplantation, increasing evidence highlights that their therapeutic benefits are significantly mediated through exosomes—nanoscale extracellular vesicles secreted by MSCs and other cell types [34]. These exosomes are enriched with bioactive molecules, including proteins, lipids, messenger RNAs (mRNAs), and microRNAs (miRNAs), and play a critical role in intercellular communication and molecular trafficking under both physiological and pathological conditions. Their cargo is dynamically modulated by cellular stress such as oxidative or thermal stimuli, enabling exosomes to influence recipient cell behavior, promote homeostasis, and mitigate tissue damage [35]. MSC-derived exosomes have emerged as key paracrine mediators in regenerative medicine, facilitating tissue repair by transferring signaling molecules that influence proliferation, differentiation, and immune responses [36]. In addition, they are being explored as diagnostic biomarkers, and recent advances in exosomal bioengineering have enabled the precise delivery of therapeutic agents, positioning exosomes as promising tools for targeted therapies [35,37]. Owing to their rich cargo of growth factors, cytokines, chemokines, and other extracellular vesicles actively shape a regenerative microenvironment. Compared to cell-based therapies, exosome-based strategies offer distinct advantages, including reduced immunogenicity, lower tumorigenic risk, and fewer ethical concerns [38,39].

## 3. Exosomes from iPSC-Derived MSCs (iMSCs)

A major breakthrough in stem cell biology is the generation of induced pluripotent stem cells (iPSCs) through the epigenetic reprogramming of somatic cells [24,40]. These iPSCs possess the ability to differentiate into multiple lineage cell types, including iPSC-derived MSCs (iMSCs) [24,40], which are a renewable, patient-specific source for autologous therapies [24]. Compared to adult MSCs, iMSCs offer enhanced proliferative capacity, greater cellular heterogeneity, and potent anti-inflammatory properties [40,41]. The regenerative potential of iMSCs has been particularly demonstrated in bone repair models. Both early- and late-passage iMSCs, as well as BM-MSCs, have shown the ability to contribute to bone regeneration when orthotopically injected into radial defects [42]. In vitro iMSCs derived from human fetal foreskin fibroblasts (HFFs) have differentiated into osteoblasts, expressed various bone morphogenic proteins (BMPs), and secreted key paracrine factors such as PDGF-AA and osteopontin. In a mini-pig model, HFF-iMSC combined with calcium phosphate granules (CPGs) resulted in significantly enhanced bone consolidation compared to CPGs alone, and performed comparably to autologous bone marrow concentrate (BMC) combined with CPGs after six weeks, suggesting iMSCs to be a promising treatment option for load-bearing bone defects [43]. Beyond their direct application, the secretomes of iMSCs, particularly their exosomes, play an equally critical role in mediating therapeutic effects [28,44]. Notably, iMSCs, regardless of the donor’s age or tissue source, acquire a rejuvenation-associated gene signature and a secretome profile similar to fetal MSCs (fMSCs) and adult MSCs (aMSCs). This rejuvenation signature, including the expression of INHBE, DNMT3B, POU5F1P1, CDKN1C, and GCNT2, is also found in pluripotent stromal cells but not in parental adult MSCs [45]. Proteomic comparison revealed that iMSC- and umbilical-cord-derived MSC (UC-MSC) secretomes are enriched with factors associated with proliferative potential and telomere maintenance, whereas secretomes from adult-tissue-derived bone marrow (BM-MSC) and adipose-tissue-derived MSCs (AT-MSCs) contain higher levels of fibrotic and extracellular matrix (ECM) proteins [46,47]. Although the application of iMSC-derived exosomes in bone regeneration remains unexplored, their similarity to conventional MSC-derived exosomes suggests that they may possess enhanced therapeutic potential due to the superior attributes of their parental cells [35,48]. Given their ability to regulate stress responses and foster tissue homeostasis, iMSC-derived exosomes represent a promising next-generation, cell-free approach for advanced bone repair and regeneration therapies. These findings underscore the superior vitality and therapeutic capacity of iMSCs compared to adult-tissue-derived MSCs.

## 4. MSC-Derived Exosomes in Bone Regeneration

Several in vitro studies have demonstrated that MSC-derived exosomes enhance the proliferation and differentiation of osteoblasts, the cells responsible for new bone formation [49,50]. These exosomes carry a wide range of bioactive cargo, including proteins, mRNAs, and microRNAs (miRNAs), and play a crucial role in cell-to-cell communication and tissue regeneration, extending beyond the action of cytokines (Table 1). Notably, exosomal miRNAs promote osteogenic gene expression while suppressing negative regulators of bone formation, thereby influencing osteoblasts’ maturation into osteocytes. Specifically, Wang et al. (2022) [51] reported that exosomal miR-21 enhances osteoblast proliferation and differentiation by modulating the TGF-β signaling pathway, which also plays a crucial role in osteocyte differentiation. Similarly, miR-29a has been shown to promote osteoblast differentiation and extracellular matrix mineralization, indirectly supporting a favorable microenvironment for osteocyte maturation [51]. In addition to osteogenesis, MSC-derived exosomes also stimulate angiogenesis, a key process for delivering oxygen and nutrients to regenerating bone tissue, thereby supporting both osteoblast and osteocyte survival and function [52,53].

These exosomes act as important mediators in bone regeneration by delivering functional cargo to target cells and regulating their proliferation, differentiation, and function [56]. Gene and proteomic analyses have revealed that exosomes are enriched in diverse proteins, including cytoskeletal elements, adhesion molecules, and transcription factors (Table 2).

Functionally, exosome-associated proteins contribute significantly to bone healing. For instance, the injection of exosomes isolated from MSC-conditioned medium improved impaired fracture healing in CD9−/− mice, which are deficient in exosome production. This suggests that non-cytokines’ cargo, especially miRNAs, play a central role in bone repair [60]. Exosomes mediate bone regeneration not only via osteogenesis (bone formation) but also through angiogenesis (new blood vessel formation). Furthermore, exosomes influence inflammation, a key component of bone healing and the pathogenesis of skeletal disorders, by modulating the local immune response [61]. Recent proteomic studies have identified specific proteins involved in fracture healing, including ribosomal proteins, ferritin, and beta-actin, offering new insights into potential therapeutic targets [62]. Exosomes can carry and deliver bone morphogenetic proteins (BMPs) and TGFβ1, which regulate bone formation. They also deliver RANK (receptor activator of nuclear factor κB) and RANKL (RANK ligand) to modulate osteoclast differentiation, an important process during bone resorption [63].

The MSC secretome comprises both soluble factors (cytokines, growth factors) and a vesicular fraction (microvesicles and exosomes), each playing a role in bone regeneration. The regenerative ability of exosomes stems from their ability to simultaneously promote osteogenesis, stimulate angiogenesis, and modulate inflammation [61,64].

In vivo studies using animal models of bone defects have further supported the bone-regenerative potential of MSC-derived exosomes. Both local and systemic administration of these vesicles has been shown to accelerate bone repair, increase bone volume, and improve the mechanical strength of newly formed bone [18,54]. These outcomes are crucial for restoring the structural integrity and long-term function of bone tissue, which is critically dependent on a well-maintained osteocyte network. Furthermore, MSC-derived exosomes help modulate the inflammatory response at the defect site, creating a more favorable environment for tissue regeneration [65,66,67]. The therapeutic efficacy of MSC-derived exosomes in bone regeneration can be further enhanced through strategies such as preconditioning MSCs (Table 3) with specific growth factors or cytokines before to exosome isolation [22,68,69]. This approach can enrich the exosomal cargo with pro-osteogenic and pro-angiogenic factors, thereby improving their regenerative potential [70]. While current preconditioning strategies primarily focus on enhancing osteoblast activity and bone formation, future efforts may target osteocyte-specific pathways to optimize outcomes in bone regeneration. Additionally, strategies include loading exosomes with therapeutic agents or engineering their surfaces to improve targeted delivery to bone tissues [71,72,73,74].

Compared to direct MSC transplantation, MSC-derived exosomes present a cell-free alternative with several advantages, including lower immunogenicity, reduced risk of tumorigenesis, and fewer off-target effects [38,88]. Their lack of surface-bound proteins also contributes to reducing immunogenicity, and their scalability and ease of storage make them highly practical for clinical applications. Altogether, exosome-based therapies hold strong promise for the safe and effective regeneration of healthy bone tissue.

## 5. Immunomodulatory Properties of MSC-Derived Exosomes

MSC-derived exosomes play a crucial role in bone regeneration by mediating communication within the skeletal microenvironment and influencing key processes such as the transition of osteoblasts to osteocytes. These vesicles deliver bioactive molecules including proteins, RNAs, and lipids that regulate the behavior of recipient cells and support bone healing and remodeling [12,89]. Through paracrine signaling, MSC-derived exosomes promote osteoblast differentiation into osteocytes, and they contribute to bone homeostasis and mineralization [11,90]. Furthermore, they enhance neovascularization and modulate local immune responses, both of which are vital for effective bone repair [89,91]. These regenerative mechanisms include transferring microRNAs and proteins that influence gene expression, cell proliferation, and lineage commitment, while also interacting with osteoclasts and osteocytes to maintain skeletal balance [89,90,92]. Despite their therapeutic promise, translating MSC-derived exosome therapies into clinical therapies remains a challenge due to complexities in exosome biology and the need for a standardized production protocol.

**Figure 2:**
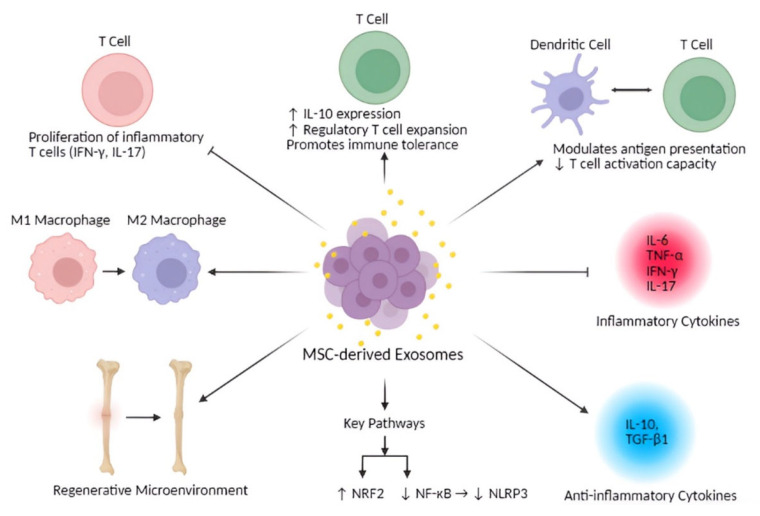
**Immunomodulatory effects of MSC-derived exosomes.** The exosomes modulate T cell responses by suppressing the proliferation of inflammatory T cells (e.g., IFN-γ and IL-17 producers) while promoting regulatory T cell expansion and IL-10 expression, enhancing immune tolerance. They also inhibit M1 macrophage polarization, dendritic cell antigen presentation, and T cell activation capacity. Key anti-inflammatory cytokines (IL-10, TGF-β1) and pathways (↑NRF2, ↓NF-κB/NLBP3) contribute to a regenerative microenvironment, countering pro-inflammatory cytokines (IL-6, TNF-α, IFN-γ, IL-17).

MSC-derived exosomes also exhibit profound immunomodulatory properties by targeting key immune cells such as macrophages, T cells, and dendritic cells, which has important implications for bone regeneration and immune-mediated bone diseases (Table 4). These exosomes can modulate macrophage polarization, shifting cells toward either pro-inflammatory (M1) or anti-inflammatory (M2) phenotypes, depending on the local microenvironment (Figure 2). This functional plasticity is crucial for resolving inflammation and promoting regeneration in conditions such as atherosclerosis and myocardial infarction and bone healing [55,93].

Exosomes derived from various MSC sources exhibit distinct immunomodulatory profiles that are critical for their therapeutic applications in treating inflammatory diseases and promoting tissue regeneration [97,98]. For instance, exosomes from human adipose MSCs have been shown to suppress T cell activation and proliferation and IFN-γ production in vitro, suggesting their potential for managing T cell-mediated inflammatory disorders [97]. In comparative studies, exosomes from canine bone marrow MSCs demonstrate greater immunomodulatory potential than those from canine-adipose-tissue-derived MSCs (cAd-MSCs). Specifically, cAD-MSCs produce higher levels of anti-inflammatory cytokines such as IL10 and TGF-β along with a richer proteomic profile of molecules involved in immune modulation and cellular communication [99].

Despite source-related differences, MSC-derived exosomes generally exhibit robust immunosuppressive functions across species. They contribute to the resolution of inflammation by inducing M2 macrophage polarization, inhibiting pro-inflammatory T cell subsets (such as Th1 and TH17), and dampening antigen-presenting cell activity [67]. Additionally, these exosomes promote the expansion of regulatory T cells (Treg) and can shift T helper cell balance from a Th1-dominant toward a Th2-dominant profile, thereby reducing inflammatory cytokine secretion (e.g., TNFα, ILβ) and enhancing anti-inflammatory mediators like TGFβ [78,98]. Importantly, the source of the MSCs significantly influences the immunomodulatory efficacy of their exosomes. While both canine adipose and bone marrow MSCs can modulate lymphocyte activity in vitro, their exosomes do not always exert equivalent effects at the same dosage, emphasizing the need for source-specific optimization in exosome-based therapies [99].

Our group has shown that iMSCs derived from periodontal ligament fibroblasts exert potent anti-inflammatory effects [40]. Moreover, iMSCs derived from urinary epithelial cells have been shown to promote Foxp3 Treg differentiation in vitro and to reduce inflammation in vivo in ischemia/reperfusion (I/R)-injury-induced retinal degeneration models by enhancing Treg populations [100]. These iMSC-derived exosomes suppress the proliferation of activated human peripheral blood mononuclear cells (PBMCs) and shift CD4 T cells from pro-inflammatory Th1 and Th17 phenotypes towards a regulatory T cell phenotype [101]. In contrast, tumor-derived exosomes can exert immunosuppressive effects that facilitate tumor immune evasion [102]. These exosomes can polarize macrophages toward immunosuppressive phenotypes and express PD-L1, which inhibits T cell activation and induces immune tolerance [102]. However, immune-cell-derived exosomes can promote T cell activation and proliferation, making them useful in cancer immunotherapy [103]. T cell modulation is also relevant in bone-related autoimmune diseases such as rheumatoid arthritis. Exosomes also influence dendritic cell function by modulating antigen presentation and T cell activation capacity [96]. Tumor-derived exosomes impair dendritic cell metabolism, thereby reducing their ability to activate T cells [104]. Moreover, dendritic cells can also influence bone metabolism through crosstalk with osteoblasts and osteoclasts [105,106]. These dual roles of exosomes in promoting immune activation or suppression are highly context-dependent and highlight the importance of source-specific exosomal profiling for therapeutic applications.

Beyond immunosuppression, MSC-derived exosomes also contribute to inflammation resolution, a key requirement for tissue regeneration. They suppress pro-inflammatory cytokines such as IFN-γ and IL-17 and promote anti-inflammatory cytokines like IL-10 [14]. In neuroinflammatory models, MSC-derived exosomes reduce IL-6 and TNF-α levels and promote M2 macrophage polarization via pathways such as NRF2/NF-κB/NLRP3, enhancing the resolution of inflammation and tissue repair [15]. Preconditioning MSCs can further enhance these effects, making their exosomes particularly effective in bone regeneration by mitigating chronic inflammation, a major barrier to healing in aging individuals [76,77,107]. MSC-derived exosomes also regulate macrophage activity by promoting the repolarization of M1 macrophages to the anti-inflammatory and regenerative M2 phenotype, which is associated with decreased inflammation and enhanced bone formation. These shifts are characterized by the reduced expression of pro-inflammatory cytokines (e.g., IL-1β, TNF-α) and the upregulation of immunoregulatory markers (e.g., IL-10, Arg1, CD206), creating a local immune environment that supports bone healing [75,77,78,82]. In addition to their immunomodulatory actions, these exosomes directly support osteogenesis and angiogenesis, both essential components of effective bone repair [60,77,107,108]. Furthermore, they facilitate the expansion of Tregs and promote immune tolerance, which has therapeutic implications in autoimmune conditions such as multiple sclerosis and inflammatory bowel disease [14,94,95].

The immunoregulatory effects of MSC-derived exosomes are largely attributed to their cytokine and microRNA content. These exosomes suppress pro-inflammatory cytokines such as IL-1β and IFN-γ while promoting anti-inflammatory cytokines like IL-10 and TGF-β1, thereby contributing to immune tolerance [16,109,110,111,112]. They also carry angiogenic growth factors, including VEGF and HGF, which support neovascularization and tissue repair [59]. He et al. (2024) found that miR-540-3p modulates dendritic cells and T cell responses through the CD74/NF-κB axis, enhancing immune tolerance and reducing graft rejection [16]. It has been reported that miRNAs such as miR-3940-5p, miR-22-3p, and miR-16-5p inhibit tumor proliferation and migration by targeting oncogenic pathways [17]. Similarly, miRNAs such as miR-21, miR-146a, and miR-181 are involved in regulating inflammatory signaling and enhancing cell survival [57,58]. However, while these molecular mediators show therapeutic promise, it is essential to consider that MSC-derived exosomes can also contribute to immunosuppression in tumor settings [102], necessitating their cautious application in cancer-related contexts. The heterogeneity of exosome cargo and the variability of immune environments underscore the need for refined, targeted therapeutic strategies. For instance, our recent studies showed that dental-pulp-stem-cell-derived exosomes (DPSC-Exo) accelerated periodontal tissue repair by converting pro-inflammatory macrophages to an anti-inflammatory response, thereby suppressing periodontal inflammation and modulating the immune response [29]. These findings highlight the potential of tailored exosome therapies in modulating local immune responses for tissue regeneration.

## 6. Non-Immunomodulatory Properties of MSC-Derived Exosomes

MSC-derived exosomes exert a multifaceted influence on bone repair beyond their immunomodulatory functions. These exosomes carry a rich cargo of bioactive molecules such as proteins, lipids, and nucleic acids that regulate various cellular processes central to tissue regeneration, including proliferation, migration, differentiation, angiogenesis, and matrix remodeling [113,114,115]. One of the key non-immunomodulatory functions of MSC-derived exosomes is the enhancement of cell proliferation and migration, both of which are essential for initiating tissue repair [116,117]. MSC exosomes have been shown to enhance the proliferation and migration of various cell types such as fibroblasts, endothelial cells, osteoblasts, and tendon stem/progenitor cells. For example, exosomes from adipose-derived MSCs promote human dermal fibroblast proliferation and migration in a dose-dependent manner, attributed to the activation of key signaling pathways including PI3K/Akt, ERK, and STAT3. These effects are further supported by the increased expression of growth factors such as hepatocyte growth factor (HGF), insulin-like growth factor-1 (IGF1), nerve growth factor (NGF), and stromal-derived growth factor-1 (SDF1), all of which contribute to tissue repair and bone healing [118]. Exosomes also promote osteogenic and tenogenic differentiation. Bone-marrow-MSC-derived exosomes have been shown to promote the tenogenic differentiation of tendon stem/progenitor cells, thereby supporting the integration of musculoskeletal tissue during bone healing [119].

Another important mechanism is the cytoprotective effect of exosomes. By reducing apoptosis and enhancing the survival of stressed cells at the fracture site, exosomes help preserve viable cell populations necessary for regeneration [113,115]. These protective effects are mediated through antioxidative pathways and the upregulation of survival-related genes. For instance, engineered MSCs that release nitric oxide (NO) show improved cell retention and viability in ischemic conditions, highlighting the role of MSC-derived exosomes in oxidative stress mitigation during early bone repair. They can significantly increase cell retention and prolong cell survival in ischemia/reperfusion-injured kidneys, partly due to the upregulation of antioxidation- and survival-related genes and the negative regulation of cell apoptosis pathways. This demonstrates a protective effect against oxidative stress, a common challenge in the hypoxic environment of early bone fracture healing. In cartilage repair, MSC exosomes mediate repair by attenuating apoptosis in target cells, a mechanism directly beneficial for the chondral phase of endochondral ossification during fracture healing [63].

Angiogenesis, the formation of new blood vessels, is vital for bone healing due to the high metabolic demands of regenerating tissue. MSC-derived exosomes enhance angiogenesis by promoting tube formation in human umbilical vein endothelial cells (HUVECs) and inducing the expression of angiogenic genes such as vascular endothelial growth factor (VEGF), basic fibroblast growth factor (bFGF), and Angiopoietin-1/2 [113,118]. Exosomes from human Wharton’s jelly MSCs also promote lymphangiogenesis via Angiopoietin-2 delivery and Prox1-mediated Akt signaling, which supports fluid homeostasis and waste clearance in the healing microenvironment [113]. In addition to supporting regeneration, exosomes regulate extracellular matrix (ECM) remodeling and fibrosis, preventing excessive scar tissue formation and promoting proper tissue remodeling, which is crucial for the organized deposition of new bone matrix [115,120]. By modulating the expression of fibrotic markers like alpha smooth muscle actin (α-SMA) and Collagen I, exosomes reduce pathological scarring and promote proper tissue architecture. For instance, human-BM-MSC-derived exosomes attenuate liver fibrosis by inhibiting the Wnt/β-catenin pathway and collagen deposition, a mechanism that may be extrapolated to prevent fibrotic non-unions in bone repair [120]. These studies highlight the critical role of exosomes beyond their immunomodulatory potential during tissue repair and regeneration.

## 7. Evidence of MSC-Derived Exosomes Promoting Osteocyte Differentiation

Numerous in vitro and in vivo studies have demonstrated that MSC-derived exosomes play a crucial role in regulating osteogenic processes, including osteoblast differentiation [121,122]. For example, mechanically strained osteocytes release exosomes containing miRNAs, such as miR-3110-5p and miR-3058-3p, which promote osteoblast differentiation in MC3T3-E1 cells [123]. Bone marrow mesenchymal stem cell (BMSC)-derived exosomes have also shown significant osteogenic potential [55,124,125]. Wang et al. (2022) [92] demonstrated that BMSC-derived exosomes increase alkaline phosphatase (ALP) activity and upregulate RUNX2 expression in osteoprogenitor cells, thereby facilitating their differentiation into osteoblasts. Supporting these in vitro findings, in vivo studies using ovariectomized mouse models demonstrated that MSC-derived exosomes improve bone formation and increase bone mass, highlighting their therapeutic potential in treating osteoporosis and osteomyelitis [92,126,127]. Furthermore, our laboratory has successfully generated iPSC-derived MSCs that differentiate into osteocytes, underscoring the potential of their exosomes to drive robust osteogenesis and support osteocyte maturation [24,40].

Beyond promoting differentiation, MSC-derived exosomes also play a key role in supporting osteocyte survival and function under stress conditions. MSC-derived exosomes have been shown to ameliorate apoptosis in osteocytes and support osteoclastogenesis. For instance, exosomes from adipose-derived mesenchymal stem cells were found to reduce osteocyte apoptosis induced by hypoxia/serum deprivation in vitro. Similarly, exosomes derived from Wharton’s-jelly-derived mesenchymal stem cells were effective in reducing osteocyte apoptosis in glucocorticoid-induced osteonecrosis in vitro [128,129]. Another study demonstrated that exosomes from mouse adipose-derived stem cells, particularly after low-level laser irradiation, suppressed hypoxia-induced osteocyte apoptosis [130]. These findings suggest a protective role for MSC-derived exosomes in maintaining osteocyte viability and function, which is crucial for preserving bone homeostasis. Research also highlights the paracrine mechanisms by which MSC-derived extracellular vesicles (EVs), including exosomes, influence bone repair. Mechanically stimulated osteocytes secrete factors, including EVs, that enhance human MSC recruitment and osteogenesis, demonstrating a reciprocal regulatory relationship wherein osteocytes influence MSCs and vice versa [131].

The maturation stage of MSCs significantly influences the functional profile of their secreted exosomes in promoting osteogenesis. The maturation of MSCs involves distinct signaling pathways that vary between the early and late stages. The early stage is characterized by rapid morphological changes and the activation of specific signaling cascades, while the late stage focuses on the maturation and functional integration of the differentiated cells. During the early stages, signaling pathways such as cAMP, wnt, and BMP guide lineage commitment, while late differentiation is governed by MAPK and TGFβ, and long noncoding RNAs, which promote cellular maturation and functional integration [132,133]. In contrast, while early-stage signaling focuses on lineage commitment, late-stage signaling emphasizes functional maturation and integration into tissue. This distinction highlights the complexity of MSCs and the need for precise regulation at different stages. For example, Wang et al. (2018) demonstrated that exosomes derived from MSCs at late-stage osteogenic differentiation enhance matrix mineralization and reinforce lineage commitment toward osteoblast maturation, which eventually become osteocytes [134]. Conversely, a study has demonstrated that early-stage MSC-derived exosomes primarily promote osteogenic commitment through the activation of the PI3K/Akt pathway [135]. Additionally, it has been demonstrated that exosomal miR-20a enhances osteogenic differentiation by targeting BAMBI, a suppressor of TGF-β signaling, thereby facilitating osteoblast and osteocyte maturation [18].

The inflammatory microenvironment plays a major role in modulating the osteogenic effects of MSC-derived exosomes. Pro-inflammatory cytokines such as TNF-α have been shown to impair osteogenesis by downregulating the Wnt/β-catenin pathway and shifting MSCs’ fate toward adipogenesis [17]. However, MSC-derived exosomes can reverse this adverse effect by delivering bioactive molecules that suppress inflammation while concurrently stimulating osteogenic differentiation [12]. Furthermore, a study has reported that exosomes from osteogenically differentiated MSCs enhance osteogenic markers such as ALP and BMP2, further promoting osteoblast lineage commitment [50]. Likewise, exosomes harvested during early osteogenesis, enriched in bone-related proteins, significantly enhance osteogenic differentiation compared to naïve extracellular vesicles (EVs) [136].

Animal studies further corroborate the regenerative potential of MSC-derived exosomes in osteocyte function and bone remodeling. In ovariectomized (OVX) rat models, iMSC-derived exosomes significantly stimulated bone regeneration and angiogenesis. Although these exosomes primary targeted bone marrow MSCs, the improved bone microenvironment indirectly benefited osteocyte populations [137]. Additionally, MSC-derived exosomes mitigate radiation-induced osteoblast and osteocyte apoptosis by restoring Wnt/β-catenin signaling and rejuvenating resident MSC function [138]. In a glucocorticoid-induced osteonecrosis model, exosomal therapy prevented disease progression, illustrating protective effects in vivo [129]. Aptamer-functionalized exosomes from BM-MSCs have shown the ability to act as a home to bone tissue, enhancing bone mass in OVX mice and accelerating fracture repair, thereby highlighting their therapeutic targeting capability for osteocytes [139]. Although osteoblast injection yielded stronger anabolic responses than MSCs or exosome therapies in some models [140], exosomes still represent a versatile and minimally invasive therapeutic alternative due to their ability to influence multiple regenerative pathways, including osteogenesis, angiogenesis, and immune modulation.

In vivo studies further support the regenerative potential of MSC-derived exosomes (Table 5). It has been demonstrated that these exosomes significantly improved bone formation and integration with implants in osteoporotic rat models, confirming their efficacy in physiological systems [18]. Moreover, the exosomes derived from younger plasma sources possess higher osteogenic potential than those from aged plasma, suggesting that donor age influences the therapeutic quality of exosomal content [141]. Despite the promising outcomes, the precise mechanisms through which MSC-derived exosomes influence osteocyte differentiation and broader bone remodeling remain an area of active investigation. The heterogeneity of exosomal cargo, influenced by the MSC source and culture conditions, and the dynamic interplay with the inflammatory milieu, present ongoing challenges. Further investigation is needed to delineate the molecular pathways governing exosome-mediated osteocyte maturation. Addressing these challenges, particularly through the development of targeted exosome delivery systems, could significantly improve the clinical utility of MSC exosome therapies for inflammatory bone diseases and skeletal regeneration. Although much of the current literature focuses on the impact of MSC-derived exosomes on osteoblast proliferation and early differentiation, the transition toward fully mature osteocytes remains a critical frontier in regenerative research [11,12,51]. Studies have shown that the successful generation of iPSC-derived MSCs capable of differentiating into osteocytes provides a strong foundation for future explorations into the role of exosomes in terminal osteocyte maturation [24,40,142]. Ultimately, the capacity of MSC-derived exosomes to modulate osteogenic differentiation while mitigating inflammation represents a promising strategy for advancing regenerative medicine, especially in the context of musculoskeletal disorders.

## 8. Therapeutic Potential and Clinical Implications

**Figure 3:**
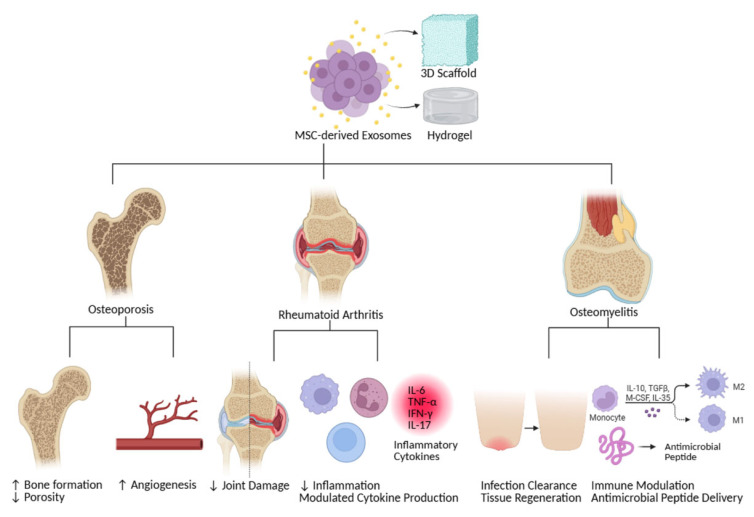
**Therapeutic potential of MSC-derived exosomes for bone diseases.** This schematic diagram illustrates the multifaceted therapeutic roles of mesenchymal stem cell (MSC)-derived exosomes in addressing various bone disorders. MSCs secrete exosomes, which can be directly administered or integrated into biomaterial scaffolds. In osteoporosis, exosomes enhance bone density and reduce fracture risk by stimulating osteoblast activity and inhibiting osteoclastogenesis. In rheumatoid arthritis, they alleviate joint damage and modulate cytokine production by suppressing inflammatory pathways. In osteomyelitis, exosomes promote tissue regeneration and modulate immune responses, possibly by delivering antimicrobial peptides and regulating inflammatory cell activity, aiding in the healing of infected bone tissues.

MSC-derived exosomes are increasingly recognized as a promising cell-free alternative to MSC transplantation for bone regeneration (Figure 3). These nanoscale vesicles encapsulate the therapeutic potential of MSCs while avoiding several risks associated with live cell therapy, such as tumorigenicity, immune rejection, and ethical concerns [38,39]. Replicating the anti-inflammatory, osteoinductive, and angiogenic functions of MSCs, exosome-based therapies offer a more controllable and safer approach for treating skeletal disorders. Their small size and intrinsic tissue tropism enable MSC exosomes to efficiently deliver therapeutic cargo—such as microRNAs, proteins, and lipids—to sites of injury and regeneration, including bone [143,144]. These features make them particularly suited for targeting the bone microenvironment. Ongoing research is focused on enhancing their bone targeting specificity by engineering their surfaces with bone-homing peptides, thereby improving their retention at sites of skeletal injury. Exosomes also exhibit excellent stability in circulation and can cross biological barriers, increasing their clinical utility for both systemic and local administration. However, several challenges must be addressed before their routine use in clinical settings. While exosomes hold immense therapeutic promise, their biosafety profile requires rigorous evaluation. For instance, certain exosomes, such as those derived from activated glial cells, have been shown to exacerbate neuroinflammation and neuronal injury raising neurotoxicity concerns in the context of neurodegenerative diseases [145]. Similarly, exosomes from mice with acetaminophen-induced liver injury induced toxicity in hepatocytes, resulting in an increase in cell mortality and inflammation [146]. Other studies have shown that exosomes from pathological sources, such as preeclampsia, can impede vascular function by delivering antiangiogenic factors to endothelial cells, further underscoring potential off-target risks [147].

In addition to biosafety considerations, technical challenges such as heterogeneity of exosomal cargo, variability in production methods, and lack of standardization in isolation and storage protocols pose significant barriers to clinical translation. The precise molecular mechanisms underlying the therapeutic effects of exosomes in bone repair, particularly their role in modulating osteoimmunology and remodeling, also remain to be fully elucidated. To overcome these challenges and optimize therapeutic outcomes, recent efforts have explored the integration of MSC exosomes with biomaterial scaffolds (Table 6). Advanced delivery systems—such as hydrogels, collagen sponges, and 3D-printed scaffolds—can enable the sustained and localized release of exosomes at the site of bone injury [65]. These scaffolds not only improve the retention and bioavailability of exosomes but also provide mechanical support and structural cues for tissue regeneration. MSC exosomes embedded in such scaffolds deliver bioactive molecules, including osteogenic miRNAs, growth factors, and immunomodulatory proteins, which promote angiogenesis, modulate inflammation, and enhance osteoblast differentiation and bone matrix deposition [11,12,51,89,134].

Importantly, these exosome-functionalized scaffolds offer an attractive alternative to cell-based therapies by overcoming issues related to immune incompatibility, regulatory complexity, and storage logistics. The use of biocompatible and customizable scaffolds allows for precise control of exosome release kinetics and provides a versatile platform adaptable to patient-specific needs. Moreover, preconditioning MSCs with osteoinductive or neurotrophic agents—such as nerve growth factor (NGF)—has been shown to enrich the exosomal cargo, enhancing both neuroregenerative and osteogenic effects [157]. This strategy may improve the maturation of osteoblasts into fully functional osteocytes and further support bone remodeling.

## 9. Challenges and Future Directions

Despite the compelling preclinical evidence supporting the osteoinductive and immunomodulatory capabilities of MSC-derived exosomes, several critical challenges hinder their clinical translation. A major limitation lies in the heterogeneity of exosomal cargo, which is profoundly influenced by the MSC source, culture conditions, and the stage of differentiation [158,159]. This variability undermines reproducibility across studies and complicates the consistency of therapeutic outcomes, particularly in bone regeneration. However, our work with iPSC-derived MSCs shows promise that these cells maintain stable characteristics and chromosomal integrity even at later passages, offering a more uniform and scalable source for exosome production [24]. Another challenge stems from the species-specific nature of bone biology research. Much of our current understanding of bone remodeling and osteocyte function is based on single-species studies, limiting the generalizability of the findings. Comparative cross-species research is needed to define conserved mechanisms and translate the findings more broadly [160].

An emerging concern is the role of cellular senescence and senescent drift in MSC cultures. Exosomes secreted by senescent cells may carry a pro-inflammatory secretome, known as the senescence-associated secretory phenotype (SASP), which can impair regenerative outcomes and induce dysfunction in surrounding cells [161]. These senescent exosomes have been implicated in the propagation of senescence signals—a phenomenon termed “senescent drift”—which can exacerbate tissue inflammation and degeneration in recipient cells, undermining the intended therapeutic benefit [162]. Rigorous screening for senescent markers in exosome-producing MSC populations and the standardization of isolation and quality control protocols are essential prior to clinical use.

Critically, the role of MSC exosomes in osteocyte differentiation remains underexplored. While numerous studies have demonstrated their effects on osteoblast proliferation and early-stage osteogenesis [49,50], the specific molecular mechanisms driving the osteoblast-to-osteocyte transition are poorly defined [11,12]. This knowledge gap is particularly significant given the central role of osteocytes in bone homeostasis, mechanotransduction, and skeletal disease pathophysiology [7]. Moreover, the inflammatory microenvironment profoundly influences exosome function. Although MSC-derived exosomes can attenuate inflammation and promote bone repair, pathological conditions such as diabetes and osteoporosis alter their bioactive content, reducing efficacy [49,54]. Future work should focus on identifying disease-induced alterations in exosomal cargo and developing engineering strategies to enhance their regenerative potential even under such pathological conditions. Understanding how microenvironments modulate exosome function will be crucial for improving therapeutic precision, particularly regarding osteocyte differentiation and survival.

Further progress also requires advanced in vivo imaging and tracking systems. Although in vivo studies have shown improved bone formation and implant integration with MSC exosome administration [18,54], the biodistribution and behavior of exosomes within the osteocyte network remain poorly understood. Innovations in high-resolution intravital microscopy and humanized bone models could provide unprecedented insights into how exosomes interact within the bone microenvironment. Coupling this with lineage-tracing tools could clarify their fate and mechanisms of action.

Another frontier involves targeted delivery strategies. Biomaterial scaffolds—such as hydrogels and 3D-printed constructs—have demonstrated potential for sustained localized exosome release [65,71]. However, ensuring compatibility between scaffold composition and exosome bioactivity remains a challenge. Future research should explore surface conjugation with bone-homing peptides and the use of stimuli-responsive biomaterials to achieve spatial and temporal precision in exosome release at sites of bone injury. Equally critical is the need to optimize manufacturing protocols for clinical-grade MSC-derived small extracellular vesicles (sEVs). This includes scalable production, high-purity isolation, and long-term storage methods [89]. Equally important is the need to standardize exosome isolation, characterization, and quantification protocols. Current methods—including ultracentrifugation, precipitation, and immunoaffinity capture—often result in inconsistent purity, yield, and functional activity [163,164]. Without universally accepted protocols, clinical scalability and inter-study comparisons remain limited. Addressing this will require coordinated, multi-institutional efforts to develop reproducible and regulatory-compliant standards for exosome manufacturing and quality control.

Lastly, the immunological context in which MSC exosomes operate adds another layer of complexity. Their immunomodulatory effects vary with MSC source, tissue of origin, and the surrounding inflammatory cues [15,17]. Unraveling the specific receptors and signaling pathways mediating interactions between exosomes and immune cell subsets within the bone niche is essential for developing context-specific, disease-targeted therapies. While preclinical studies in small animal models demonstrate robust bone regenerative effects, large animal studies are required to evaluate efficacy, safety, and dose-responsiveness under physiologically relevant conditions [107,165]. The transition to clinical trials also necessitates addressing regulatory concerns regarding exosome safety, reproducibility, and off-target effects [166].

Looking forward, future research should prioritize three interconnected goals:

(1) Dissecting the molecular pathways through which MSC exosomes influence osteocyte differentiation and function.
(2) Establishing standardized, scalable protocols for exosome production, purification, and characterization.
(3) Innovating delivery systems—such as engineered scaffolds and targeting ligands—to maximize therapeutic precision.

Interdisciplinary collaboration across stem cell biology, biomaterials engineering, immunology, and regenerative orthopedics will be vital to overcome these challenges. Addressing them effectively could revolutionize musculoskeletal therapy, enabling the development of less invasive, more targeted, and highly personalized treatments for a wide range of bone-related disorders. Nevertheless, several key questions remain unresolved. Identifying the optimal scaffold composition, loading strategies, and release profiles is essential for clinical translation. Additionally, understanding the interactions between exosomal cargo and resident bone cells at the molecular level will help refine therapeutic designs. Standardizing manufacturing processes for exosome isolation, characterization, and integration into biomaterials is another critical step toward regulatory approval and large-scale clinical deployment. In summary, MSC-derived exosomes, particularly when combined with bioengineered scaffolds, represent a cutting-edge approach to bone regeneration. Their immunomodulatory, osteogenic, and angiogenic properties—coupled with their safety and adaptability—highlight their strong translational promise. With the continued refinement of delivery systems and mechanistic understanding, MSC-derived-exosome-based therapies are poised to become a powerful tool in the clinical management of bone diseases.

## 10. Conclusions

Mesenchymal stromal cells (MSCs) and their derived exosomes represent a promising frontier in regenerative medicine, particularly for their versatile roles in bone regeneration and immunomodulation. These naturally occurring nanocarriers facilitate intercellular communication by modulating osteoblast differentiation, osteocyte maturation, angiogenesis, and key immune responses within the bone microenvironment. Compared to traditional cell-based therapies, exosome-based approaches offer several advantages, including reduced immunogenicity and improved safety profiles. However, significant challenges remain—most notably the need to standardize exosome production and characterization, address cargo heterogeneity, and enhance targeted delivery strategies. Future research must prioritize the elucidation of molecular mechanisms, the development of robust in vivo models, and the integration of interdisciplinary expertise spanning stem cell biology, biomaterials, and immunology. These efforts will be critical for advancing the clinical translation of MSC-derived exosome therapies and hold the potential to transform the treatment landscape for a wide range of musculoskeletal disorders.

## Figures and Tables

**Table 1:** Effect of MSC-derived exosomes in bone regeneration.

Effect		Specifications	Citation
Promotion of Osteogenesis	Enhance osteoblast proliferation and differentiation	Deliver specific microRNAs (miRNAs) that promote osteogenic markers and inhibit negative regulators of bone formation. For instance, miR-21 enhances osteoblast proliferation and differentiation by targeting the TGF-β signaling pathway. miR-29a promotes osteoblast differentiation and extracellular matrix mineralization.	[49]
			[50]
			[51]
	Influence osteoblast maturation into osteocytes	Exosomes deliver specific microRNAs (miRNAs) that influence the maturation of osteoblasts into osteocytes.	[49]
			[50]
Stimulation of Angiogenesis	Promote formation of new blood vessels	Crucial for supplying nutrients and oxygen to regenerate bone tissue and develop osteocytes within that tissue.	[52]
In Vivo Bone Regeneration	Accelerate bone healing, increase bone volume, and improve mechanical strength	Animal models of bone defects have demonstrated this in response to local or systemic administration. Crucial for restoring structural integrity and long-term functionality of bone tissue	[18]
			[54]
Modulation of Inflammatory Response	Create a favorable environment for tissue regeneration	MSC-derived exosomes can modulate the inflammatory response in the bone defect site	[55]

**Table 2:** Exosomal cargo and their roles in bone regeneration.

Bioactive Cargo	Function	Target Factors	Signaling Pathway	MSC Type	Citation
miR-21	Enhances osteoblast proliferation and differentiation	TGF-β modulators	TGF-β/Smad	BMSC	[51,57,58]
miR-29a	Promotes osteoblast differentiation and ECM mineralization	RUNX2, COL1A1	Wnt/β-catenin	BMSC	[51]
miR-20a	Enhances osteogenic differentiation	BAMBI	TGF-β/Smad	BMSC-derived sEVs	[18]
miR-540-3p	Enhances immune tolerance and reduces graft rejection	CD74	NF-κB pathway	miR-540-3p-overexpressing MSCs	[16]
miR-16-5p	Suppresses inflammation and tumor progression	Cyclins, BCL2	Apoptosis, NF-κB	MSC-derived exosomes	[17]
miR-146a	Suppresses inflammation, promotes osteogenic survival	TRAF6, IRAK1	NF-κB inhibition	MSC-derived exosomes	[57,58]
miR-181	Regulates inflammation and supports osteogenesis	Notch regulators	NF-κB, Notch	MSC-derived exosomes	[57,58]
Pro-inflammatory cytokines (IL-1β, IFN-γ)	Inhibit osteogenesis and increase bone resorption	Osteoblasts, immune cells	NF-κB, MAPK	Present in some MSC-derived exosomes (low levels)	[16]
Anti-inflammatory cytokines (IL-10, TGF-β1)	Promote osteogenesis and suppress inflammation	Immune cells, osteoblasts	TGF-β/Smad, NRF2	AD-MSC, UC-MSC, iMSC	[16]
VEGF	Promotes angiogenesis and bone vascularization	VEGFR	PI3K/Akt	UC-MSC, iMSC	[59]
HGF	Enhances angiogenesis and tissue repair	c-Met	MAPK/ERK	MSC-derived exosomes	[59]
BMPs	Induce osteogenesis	BMP receptors	BMP/Smad pathway	iMSC, BM-MSC	[43]

**Table 3:** Preconditioning strategies to enhance the immunomodulatory and regenerative effects of MSC-derived exosomes.

Preconditioning Method	Specific Strategy/Stimulus	Enhanced Effects	Citation
Inflammatory Cytokine Stimulation	TNFα Preconditioning	Suppresses IL-1β and iNOS and increases Arg1 and CD206 in macrophages; enhances bone formation	[75]
	Pro-inflammatory Cytokines (TNFα, IL17A)	Mimics inflammatory conditions to boost immunosuppressive exosomal content	[76,77]
	IL-1β and TNF-α Exposure	Reduces IL-1β and TNF-α and increases IL-10 and other anti-inflammatory cytokines	[78]
Hypoxia Preconditioning	Low Oxygen Conditions	Enhances exosome yield, angiogenesis, and anti-inflammatory potential	[79,80,81]
	Ischemic Preconditioning	Reduces TNFα and neutrophils, increases IL-10, and improves recovery in lung injury models	[82]
	Secretome Modulation	Boosts MSC survival, migration, and regenerative paracrine activity	[83,84,85]
Mechanical Stress	Mechanical Strain (Exercise Mimetic)	Increases exosome production and enhances myogenic differentiation and cell proliferation	[86]
	Mechanical Stress-Induced Cargo Modulation	Alters exosome miRNA cargo and modulates BMP signaling	[86,87]

**Table 4:** Immunomodulatory effects of MSC-derived exosomes on immune cells.

Immune Cell Type	Experimental Model	Observed Immunomodulatory Effects	Citation
Macrophages	In Vitro	Induce either pro-inflammatory or anti-inflammatory phenotypes depending on the microenvironment	[93]
	In Vivo	Promote the M2 macrophage phenotype in neuroinflammatory models	[15]
Macrophages (DPSC-Exo specific)	In Vivo	Facilitate the conversion of macrophages from a pro-inflammatory to an anti-inflammatory phenotype, thereby suppressing periodontal inflammation	[29]
T cells	In Vitro	Suppress proliferation of inflammatory T cells (producing interferon-γ and IL-17)	[14]
T cells	In Vitro	Enhance IL-10 expression	[94]
T cells	In Vivo	Encourage regulatory T cell expansion and immune tolerance	[95]
Dendritic cells	In Vitro	Alter antigen presentation and T cell activation capacity	[96]
Dendritic cells	In Vitro	miR-540-3p modulates dendritic cells via the CD74/NF-κB pathway	[16]

**Table 5:** Preclinical studies of MSC-derived exosomes in bone regeneration models.

Disease/Experimental Model	Exosome Origin	Key Findings	Citation
**In Vitro Studies**			
Hypoxia/serum-deprived osteocytes	Adipose MSCs (ADSCs)	Exosomes reduce osteocyte apoptosis	[128]
Glucocorticoid-induced osteonecrosis model	Wharton’s jelly MSCs (WJ-MSCs)	Exosomes ameliorate osteocyte apoptosis	[129]
Hypoxia-induced apoptosis	Mouse-adipose-derived MSCs (ADSCs), post-laser	Exosomes reduce osteocyte apoptosis under stress	[130]
Mechanical stimulation	Osteocyte-derived EVs	miR-3110-5p and miR-3058-3p promote osteoblast differentiation	[123]
Osteoblast precursor culture	Bone marrow MSCs (BMSCs)	Increased ALP and RUNX2 expression, promoting osteogenesis	[92]
**In Vivo Studies**			
Ovariectomy-induced osteoporosis	hiPSC-MSCs	Promote bone regeneration and angiogenesis	[137]
Glucocorticoid-induced osteonecrosis	MSCs	Prevent disease progression and support osteocyte survival	[129]
Femur fracture and OVX model	BM-MSCs with aptamer-exosomes	Improve bone mass and target bone delivery	[139]
Bone defect model in osteoporotic rats	UC-MSCs/plasma exosomes	Enhance bone formation and implant integration	[18,141]

**Table 6:** Scaffold types and properties for MSC-derived exosome delivery in tissue regeneration.

Scaffold Type	Material Composition	Biodegradability	Mechanical Strength	Surface Characteristics	Citation
Hydrogels	Naturally derived or synthetic polymers	Enable sustained release of exosomes	Allow for controlled and prolonged release of biomolecules	Water-swollen networks	[148,149,150]
Mineral-Doped Poly(L-lactide) Acid Porous Scaffolds	Polylactic acid (PLA), calcium silicates (CaSi), dicalcium phosphate dihydrate (DCPD)	Porosity decreases after 28 days in simulated body fluid; bioresorbable	Pores range from 10 to 30 µm in diameter	Circular and elliptic pores; dynamic surface that creates a bone-forming microenvironment; exosomes are easily entrapped on the surface	[151]
3D-Printed Composites	Poly(l-lactide) (PLA)	Often biodegradable, with properties that can be tuned; zein-containing scaffolds show dose-responsive improvement in degradation rate	Mechanical strength can be enhanced by incorporating materials like multi-walled carbon nanotubes (MWCNTs) in poly(L-lactide); addition of pristine graphene improves mechanical performance; Young’s modulus and yield stress can be enhanced	Can have specific architectures and porous structures; can be coated with materials like poly(dopamine) and fibrin gel for bioactivity; can exhibit better cell affinity with components like zein	[49,152,153]
Acellular Extracellular Matrices (ECMs)	Decellularized tissues, preserving natural tissue architecture	Biodegradable	Provide structural support	Mimic the native extracellular environment, promoting cell adhesion and growth	[154]
Hyaluronic Acid (HA)	Natural polysaccharide	Biodegradable	Can be formulated to have various mechanical properties	Highly biocompatible, often used in hydrogels	[148]
Polymer-Based Elastomeric Membranes	Polycaprolactone (PCL)	Biodegradable, with degradation rates that can be matched to tissue regeneration; zein can increase biodegradability	Can be designed with favorable mechanical properties; PCL/pristine graphene scaffolds show improved mechanical performance; PCL/zein composite inks can significantly improve Young’s modulus and yield stress	Exosomes are easily entrapped on the surface; electrospun nanofibers offer a high surface-to-volume ratio; hydrophobicity can be reduced by adding pristine graphene	[150,153,155,156]

## Data Availability

The original contributions presented in the study are included in the article, further inquiries can be directed to the corresponding author.
